# The impact of autophagy during the development and survival of glioblastoma

**DOI:** 10.1098/rsob.200184

**Published:** 2020-09-02

**Authors:** Joanne E. Simpson, Noor Gammoh

**Affiliations:** Cancer Research UK Edinburgh Centre, Institute of Genetics and Molecular Medicine, University of Edinburgh, Crewe Road South, Edinburgh EH4 2XR, UK

**Keywords:** autophagy, brain tumour, cancer, glioblastoma, therapy, receptor tyrosine kinase (RTK)

## Abstract

Glioblastoma is the most common and aggressive adult brain tumour, with poor median survival and limited treatment options. Following surgical resection and chemotherapy, recurrence of the disease is inevitable. Genomic studies have identified key drivers of glioblastoma development, including amplifications of receptor tyrosine kinases, which drive tumour growth. To improve treatment, it is crucial to understand survival response processes in glioblastoma that fuel cell proliferation and promote resistance to treatment. One such process is autophagy, a catabolic pathway that delivers cellular components sequestered into vesicles for lysosomal degradation. Autophagy plays an important role in maintaining cellular homeostasis and is upregulated during stress conditions, such as limited nutrient and oxygen availability, and in response to anti-cancer therapy. Autophagy can also regulate pro-growth signalling and metabolic rewiring of cancer cells in order to support tumour growth. In this review, we will discuss our current understanding of how autophagy is implicated in glioblastoma development and survival. When appropriate, we will refer to findings derived from the role of autophagy in other cancer models and predict the outcome of manipulating autophagy during glioblastoma treatment.

## 1. Introduction to glioblastoma

Glioblastoma, a grade IV astrocytoma, is the most common and aggressive type of primary brain tumour in adults. Gene expression analyses of patient-derived tumour cells revealed three distinct glioblastoma subtypes, classical, proneural and mesenchymal, which are classified based on their molecular genotypes [1]. Recently, these molecular subtypes have been found to associate with different cellular states identified by transcriptomic analyses [2]. Neural-progenitor-like and oligodendrocyte-progenitor-like states are enriched in cells associated with the proneural molecular subtype, while the astrocyte-like and mesenchymal-like states coincide with the classical and mesenchymal subtypes, respectively [2].

All molecular subtypes of glioblastoma are driven by the gain-of-function of receptor tyrosine kinases (RTKs) and/or the loss of tumour suppressor activities (including *PTEN*, *TP53*, *NF1* and *CDKN2a*) [3]. These events lead to the overactivation of the mitogen-activated protein kinase (MAPK) and phosphatidylinositol-3-kinase (PI3K) pathways, which stimulate cell growth and proliferation. The classical subtype is distinguished by alterations causing hyperactivation of the RTK EGFR, with the most frequent variation being the expression of *EGFRvIII*, a truncated mutant that lacks the extracellular ligand-binding domain and signals constitutively in the absence of growth factors [3]. In comparison, the overexpression of the RTK *PDGFRA* is associated with the proneural subtype, whereas the loss of *NF1* is linked to the mesenchymal subtype [3]. Nevertheless, cells derived from the same patient can harbour mutations in multiple RTKs and molecular subtypes [4,5], resulting in intra-tumoural heterogeneity and decreased patient survival [6].

The ability of tumours to switch from one subtype to another has also been observed in glioblastoma, with the underlying molecular mechanism largely unknown. Mounting evidence indicates that a pool of glioblastoma stem cells (GSCs) are a potential causative source [7]. Glioblastoma tumours contain GSCs that express varying stemness gene signatures thus providing another layer of inter- and intra- tumour diversity [8]. GSCs may also contribute to tumour dormancy, recurrence and resistance to therapy [7].

The current standard of care for glioblastoma patients is surgical resection followed by treatment with the chemotherapeutic agent, temozolomide [9,10]. However, the relapse of glioblastoma tumours is inevitable and treatment resistance develops such that the median survival of patients from the time of diagnosis is approximately 15 months. Variations in median survival exist between patients harbouring a predominant glioblastoma subtype, with mesenchymal tumours exhibiting the worst prognosis (11.5 months) compared to classical and proneural tumours (14.7 and 17.0 months, respectively) [1].

One potential explanation for the molecular subtype-dependent variation in the survival of glioblastoma patients is the differences in the tumour immune microenvironment. Approximately, one-third of the glioblastoma tumour mass is composed of innate immune tumour-associated macrophages (TAMs) that have mostly infiltrated from the peripheral immune system [11,12]. These TAMs express an anti-inflammatory ‘M2’ phenotype, which is associated with tumour cell immune evasion [12,13]. The recruited macrophages are enriched in the mesenchymal subtype, in comparison to the proneural and classical subtypes, which may contribute to the enhanced resistance to treatment observed in this subclass [1].

Irrespective of the underlying molecular mechanisms contributing to enhanced patient survival, glioblastoma remains a cancer of a high mortality rate [14]. The challenge in treating glioblastoma begins with the inability to surgically remove the entire tumour mass due to its diffuse nature and penetration into normal brain tissue. Glioblastoma cells enriched in mesenchymal subtype markers have the most invasive phenotype in comparison to the other subtypes [15]. In addition, acquired resistance to therapy is frequent and has been attributed to intra-tumoural heterogeneity and subtype switching upon tumour recurrence, which occurs in almost 50% of relapsed glioblastoma [1].

The aggressive nature of glioblastoma, including unconstrained growth, invasion into the normal brain parenchyma and resistance to therapy, indicates that glioblastoma cells have developed mechanisms to survive cell stress and proliferate under restrictive conditions. It is therefore important to understand cellular survival mechanisms used by glioblastoma cells in order to develop new treatments that can effectively target the tumour. Autophagy is one such pathway that is upregulated in cancer cells in response to stress. This review aims to explore what is known in the literature regarding the role of autophagy during the development and survival of glioblastoma cells. When appropriate, we will discuss the role of autophagy in other cancers and the potential applications to glioblastoma. However, we will first introduce the current models used to study glioblastoma and their drawbacks in order to better grasp the limitations of studying the role of autophagy in this aggressive cancer.

## 2. Limitations of current models of glioblastoma

The ideal model of glioblastoma should mimic various aspects seen in patients including molecular heterogeneity, interaction with stromal cells, invasion and exposure to growth restrictive conditions. To date, no individual model can recapitulate all these aspects. Here, we will briefly outline the advantages and limitations of the currently available models to study glioblastoma (recently reviewed in [16]).

### 2.1. Cell culture models

Cell culture models provide the flexibility to enable in-depth examination of molecular details and imaging analyses in a simplified and controlled manner. This comes at a cost of excluding the contribution of the tumour microenvironment and the underlying molecular complexity [17]. In addition, artificially altering metabolic properties by supplementing growth media with serum, growth factors and excess nutrients are common practices in cell culture models that may affect various cellular properties [18]. Furthermore, cell line divergence over time, either through the accumulation of new mutations or cell type-specific selection, highlights the need to carefully monitor molecular changes of cultured cells [16]. This was also shown in some cases whereby the injection of molecularly defined glioblastoma patient cells into mice showed divergence from the original molecular subtype [19], thereby confirming previous findings that some molecular signatures (such as the proneural subtype) are more transcriptionally stable [20].

Culture conditions are likely to influence the maintenance of original cell identity. Recent studies have shown that culturing glioblastoma patient-derived stem cells under serum-free stem cell conditions in monolayers is more likely to preserve long-term stem cell properties in comparison to cells grown in neurospheres [18,21–23]. Importantly, when intracranially injected into mice, these GSC lines can form tumours that are histopathologically similar to glioblastoma and can therefore be used for drug screening due to their stability and expandability in culture [23]. Using this monolayer culture method, both mouse and human neural stem cells (NSCs) can be easily manipulated by CRISPR/Cas9 technology to introduce tumour-associated oncogenic mutations that model glioblastoma [24]. Tumour-initiating mouse NSCs can be derived and re-implanted in the same animal species, therefore enabling tumour development in the context of an intact immune microenvironment.

### 2.2. Organoids

Three-dimensional organoids are an emerging novel model system to study glioblastoma. Organoids were initially grown from dissociated patient-derived GSCs that can infiltrate and proliferate in human cerebral organoids [25]. However, these models are labour-intensive and require prolonged periods to grow. More recently, organoids have been generated by propagating small sections from whole tumours in a defined culture media [26]. This is advantageous because undissociated glioblastoma sections can retain their heterogeneity, micro-vasculature, certain components of the tumour microenvironment, and hypoxic gradients [26]. These are attractive traits that allow the rapid generation of organoids that can be used for extensive drug screening [17] and testing immunotherapies [26]. However, these organoids may not be easily genetically manipulated, thus limiting their utility.

### 2.3. Mouse models

Mouse models of glioblastoma provide an important tool to investigate the role of the tumour microenvironment, the interaction of tumour cells with stromal cells and the vasculature, and tumour cell invasion into the brain parenchyma. Brain tumours that resemble glioblastomas can be induced in mice using genetically engineered mouse models (GEMMs) or by injecting animals with the tumour-initiating mouse-or patient-derived cells. The different glioblastoma mouse models have been comprehensively reviewed in [16].

A well-known GEMM of glioblastoma is the replication-competent avian sarcoma-leukosis virus LTR splice acceptor/tumour virus A (RCAS/TVA) model. This model has been engineered to express the avian virus receptor TVA in glial progenitor cells in order to allow the delivery of transgenes upon viral injection [27]. Particular benefits of this model are examining the mechanism of tumour initiation in an immune-intact animal as well as the flexibility of modifying the viral inserts. However, this model is restricted by the size of the viral cargo [28], an aspect overcome in more recently developed virally induced glioblastoma models [29]. Additional transgenic glioblastoma models exist but are limited by the requirement of extensive animal breeding [30,31]. Although GEMMs do not model the genetic heterogeneity and complexity observed in human tumours, they do provide insights into the role of the tumour immune microenvironment and interaction with stromal cells. Parallel studies using human cells injected into immune-compromised mice or mouse cells in syngeneic immune-competent animals are likely to provide a more comprehensive picture into the complexity of glioblastoma growth and response to treatment.

## 3. Overview of autophagy

### 3.1. Proteins involved in the autophagy pathway

Autophagy is a catabolic process that involves the engulfment of cytoplasmic components in double-membrane vesicles, called autophagosomes, which can fuse with lysosomes for the degradation and recycling of their contents to provide metabolic substrates. Autophagy occurs constitutively in cells at basal levels to maintain homeostasis through the turnover of damaged organelles and unwanted cellular material [32]. In periods of cell stress, such as nutrient starvation, hypoxia, DNA damage and pathogen infection, autophagy is upregulated in order to allow cells to adapt to the environmental changes and restore cell function [32].

**Figure 1.**
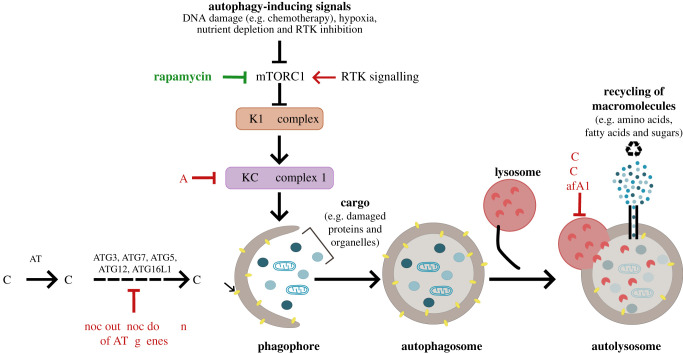
Schematic depicting an overview of the autophagy pathway and mechanisms used to manipulate autophagy. Activation of autophagy occurs upon the inhibition of the mammalian target of rapamycin (mTORC1) in response to cell stress signals, such as low nutrient supply and genotoxic stress. Autophagy activation can be achieved by mTORC1 inhibition by rapamycin or receptor tyrosine kinase (RTK) inhibition. This initiates a cascade of ATG protein complexes, beginning with the activation of the ULK1 and PI3KC3-C1 complexes and phagophore formation. PI3KC3-C1 activity can be chemically inhibited by 3-methyladenine (3-MA). Elongation and closure of the phagophore results in autophagosome maturation and requires core ATG proteins, including ATG3, ATG4, ATG7, ATG5, ATG12 and ATG16L1, that can be genetically targeted to impede this maturation step. The final stage of the pathway involves the fusion of autophagosomes with lysosomes forming autolysosomes and resulting in the degradation of the cellular cargo by lysosomal hydrolases. The degradation products (including amino acids, fatty acids and sugars) are recycled and can be used to supply cellular metabolic needs. Chloroquine (CQ), hydroxychloroquine (HCQ) and bafilomycin A1 (BafA1) are chemical inhibitors of lysosomal acidification that consequently block autophagic flux and lead to the accumulation of autophagosomes in cells.

Several autophagy-related (ATG) protein complexes are required for autophagosome biogenesis. The activation of the Unc-51-like kinase 1 (ULK1) complex can initiate autophagy during periods of nutrient and energy depletion. This can occur upon the inactivation of the mammalian target of rapamycin (mTOR) complex 1 (mTORC1) in response to low nutrient levels or activation of the AMP-activated protein kinase (AMPK) in response to energy depletion [33]. The ULK1 complex subsequently triggers the activity of the PI3K class III-complex 1 (PI3KC3-C1) containing ATG14L1, Beclin 1, Vps34 and p150, which is required to generate PI(3)P on the growing autophagosomal membrane [32]. The elongation and maturation of the autophagosome require the lipid conjugation of ATG8 protein family members (including the microtubule-associated protein 1A light chain 3 (LC3) subfamily members LC3A, LC3B and LC3C) on autophagosomal double membranes [34]. Autophagosome maturation is required for their efficient fusion with lysosomes and relies on the activities of ATG7, ATG3 and the ATG16L1-ATG5-ATG12 complex [34]. The degradation products are then recycled back to the cell, thus providing essential nutrients and energy supply to support cell growth (figure 1).

Knockout of core *Atg* genes in mice results in the loss of neonatal survival thereby revealing a vital role for autophagy during mammalian development [35]. Interestingly, the neonatal lethality in *Atg5*-deficient mice is rescued by re-expressing *Atg5* in neurons, indicating that autophagy plays an essential role in brain development [36]. Whole-body inhibition of autophagy in adult animals or neuron-specific deletion of *Atg5* or *Atg7* also resulted in neurodegeneration phenotypes in mice, further highlighting the role of autophagy in maintaining neuronal homeostasis [37–39]. The underlying mechanism for the role of autophagy in neurons is largely unknown but is probably related to its role in clearing protein aggregates. Autophagy is also involved in maintaining the function of various organs whereby mice with genetic inhibition of autophagy throughout the body, excluding the brain, also exhibited failure of various organs, ageing phenotypes and reduced lifespan [36,40]. While a crucial function for autophagy has been described in maintaining the health of the nervous system and various organs, its role during the development of cancers within the nervous system remains a largely unexplored area.

Unlike the neonatal lethality phenotype observed upon the knockout of core *Atg* genes in mice, the knockout of some autophagy players results in embryonic lethality [41–43]. This suggests that, in addition to their role in canonical autophagy (described above), these genes potentially have other functions. Of these gene products, Beclin 1 has been shown to play an additional role in endocytic trafficking by forming part of the PI3KC3 complex 2 (PI3KC3-C2) along with UVRAG, Vps34 and p150 [44,45]. Beclin 1 has been frequently associated with the development of various cancers [41,46]. However, whether the tumour-suppressive activities of Beclin 1 require its autophagic or endocytic functions remain to be further explored.

Several ATG proteins, including ATG3, ATG5 and ATG7, are also required for lipidation of ATG8 family members on single-membrane vesicles, such as phagosomes and perturbed endosomes. The functional relevance of this non-canonical ATG8 lipidation on single-membrane vesicles may be distinct from its role during autophagosome biogenesis and can be triggered by various stimuli, including pathogen infection and treatment with lysosomotropic agents [47–49]. This suggests that inhibiting the activities of core autophagy players may affect additional cellular processes. Therefore, the interpretation of the commonly used autophagy assays relying on ATG8 lipidation (especially during drug treatment) may require careful investigations. Interestingly, certain ATG complexes, such as the ULK1 complex, are dispensable for the non-canonical lipidation of ATG8 proteins and may be used as tools to distinguish canonical (double-membrane) from non-canonical (single-membrane) ATG8 lipidation.

## 3.2. Monitoring autophagy and chemical modulators

Assays to monitor autophagy mainly rely on measuring protein levels and modifications as well as cellular localization [50]. These assays should factor in the dynamic nature of autophagy that involves the initial formation of vesicles followed by the degradation of their contents, both steps being crucial for the completion of autophagic flux. The two most commonly used markers to monitor autophagy are p62, a ubiquitin-binding protein that targets cellular cargo to autophagosomes, and membrane-bound lipidated LC3 (LC3-II), which forms following the lipidation of cytosolic LC3 (LC3-I). Reduced or increased p62 levels are indicative of high or low autophagy levels, respectively. On the other hand, autophagy induction stimulates the formation of LC3-II (separated from LC3-I by a change in migration on SDS-PAGE or by cytoplasmic puncta formation) which is eventually degraded by the action of lysosomal hydrolases. Reduced LC3-II levels can indicate enhanced autophagic activity resulting in accelerated lysosomal degradation, but it may also correspond to reduced autophagosome formation and lipidation. Conversely, enhanced LC3-II protein levels could imply an increase in autophagosome biogenesis, or it may be indicative of a block in autophagosome-lysosome fusion.

To accurately measure autophagic flux, careful assays are required to induce autophagy and concurrently block the degradation step. Autophagy can be stimulated following mTORC1 inhibition by nutrient starvation or with chemical agents such as rapamycin, while blocking the lysosomal degradation of autophagosome contents is achieved using inhibitors of lysosomal acidification, such as bafilomycin A1 [51] and chloroquine (CQ) [52] (figure 1). The absence or increased accumulation of LC3-II in this setting is indicative of low or high autophagy levels, respectively. This flux assay can be used to determine autophagy inhibition by genetic or chemical targeting. Interestingly, CQ has been used in the clinic for over 50 years as a treatment of malaria while its less toxic derivative hydroxychloroquine (HCQ) is used in the treatment of the autoimmune diseases rheumatoid arthritis and systemic lupus erythematosus [53]. In addition, both CQ and HCQ can induce ATG8 lipidation on single membranes [48]. The contribution of this non-canonical autophagy activation or inhibition of lysosomal degradation during the therapeutic response to these drugs remains to be elucidated. Unlike CQ treatment, which inhibits later stages of autophagy by targeting lysosomal degradation [53], chemical inhibition of early stages of autophagy can be achieved using the Vps34 inhibitor 3-methyladenine (3-MA) or ULK1 kinase inhibitors, although toxic effects have been reported due to their modulation of additional pathways [54].

Given the dynamic nature of autophagosome biogenesis, it is challenging to measure autophagy *in vivo* and in human patients. RNA expression of *ATG* genes has also been extensively used as a readout for autophagy, but changes in transcription or protein levels do not necessarily reflect alterations in ATG protein activity. As a result, additional readouts for autophagy, such as post-translational modifications of ATG proteins [55], are needed to provide more reliable assessments of autophagic activity.

## 4. Brief overview of the involvement of autophagy in cancer

Autophagy has fundamental roles in maintaining cell health. In terms of oncogenic transformation, autophagy can play a role in preventing oxidative stress and subsequently DNA damage and cell transformation. This tumour-suppressive role of autophagy is supported by early studies that showed increased development of spontaneous, potentially benign, lung and liver tumours and lymphomas in mice deleted of autophagy genes *Beclin 1*, *Atg7* and *Atg5* [41,46,56]. Interestingly, a reversible RNAi mouse model that targets *Atg5* expression showed that, while extended autophagy inhibition can accelerate ageing, reactivating autophagy after a period of inhibition can cause increased frequency of spontaneous tumour development [40]. This suggests that while autophagy functions in preventing tumour-inducing cell damage, it may also have a role in promoting tumour growth [40]. Indeed, there is mounting evidence indicating that autophagy can affect various aspects of cancer cell growth including metabolic supply, immune evasion and response to treatment [57]. How autophagy can implicate the development and survival of brain tumours are still areas of open research where predictions can be drawn based on studies modelling various types of cancers.

Many solid tumours undergo metabolic rewiring to enable their unconstrained growth in nutrient-deprived microenvironments [58]. Recycling of cellular components (including amino acids, fatty acids and glucose) upon lysosomal degradation during autophagic flux has been shown to supply the metabolic demand of oncogenic *KRAS*- and *BRAF*-driven lung tumours [59–62]. In addition, the selective degradation of defective mitochondria by autophagy (a process known as mitophagy) might play a vital role in maintaining mitochondrial metabolism in cancer cells and support cell growth [60]. Mouse models of melanoma and pancreatic ductal adenocarcinoma (PDAC) have demonstrated that autophagy activation in stromal cells can also contribute to tumour growth by supplying metabolic substrates [63,64].

The role of autophagy during cancer treatment has been extensively studied using both animal models and cultured cells. Autophagy can be induced by a wide range of anti-cancer drug treatments. This can occur directly, for example, following mTORC1 inhibition by rapamycin [65], or release of Beclin 1 from negative regulation by EGFR using tyrosine kinase inhibitors, such as erlotinib [66]. Alternatively, autophagy may be indirectly activated by drug treatment in response to DNA damaging agents [67], or changes in metabolism (as seen following selective pharmacological inhibition of ERK in PDAC cell lines) [68]. In both cases, it is widely accepted that autophagy can help support tumour cell survival during therapy with the underlying mechanism being largely unknown and probably context dependent. In some cases, however, autophagy has been suggested to exhibit a cell death-promoting property. It is possible that the delicate balance between cell survival and death could be tipped by autophagy overactivation as a last desperate attempt to survive causing unendurable digestion of cytoplasmic proteins and organelles [69]. Indeed, the presence of autophagic structures in dying cells has been noted for many years, hence the emergence of the concept ‘death with autophagy’. Yet, although there is evidence demonstrating that autophagy can enhance the effect of some cancer drugs, autophagy upregulation in response to tumour therapies is mostly thought to be protective to tumour cells, favouring their survival and thus promoting treatment resistance [67].

Based on promising findings from pre-clinical studies, several clinical trials have been initiated to investigate the effect of concurrently inhibiting autophagy during cancer treatment [70]. Blocking the last stage of the autophagy pathway using CQ or HCQ is currently the only approved mechanism of inhibiting autophagy in humans [70]. This strategy has been shown to be effective in overcoming resistance to the *BRAF^V600E^* kinase inhibitor (vemurafenib) and restoring its efficacy in treating *BRAF^V600E^*-driven brain tumours [71,72]. More recently, pre-clinical studies demonstrated that autophagy inhibition sensitizes *KRAS*-driven PDAC to pharmacological suppression of the RAS/RAF/MEK/ERK signalling pathways [68,73] and have paved the way for a clinical trial examining the combined treatment with HCQ and the MEK1/2 inhibitor trametinib in PDAC patients [74].

Overall, the role of autophagy in cancer is multifarious, varying from being a tumour suppressing mechanism to supporting tumour growth and survival. This may reflect tumour type- and stage-specific functions of autophagy or diverse effects of inhibiting early stages versus late stages of the pathway (for example, Vps34 inhibitors versus lysosomal inhibitors). Interestingly, an oncogene-dependent effect of autophagy during tumour growth has been described in *KRAS^G12D^*-driven PDAC and non-small cell lung carcinoma (NSCLC) models, whereby autophagy loss reduced tumour growth but not when *p53* was additionally deleted [59,75]. Altogether, various considerations are required when studying the role of autophagy in cancer, including the use of model systems and oncogenic combinations, the specificity of autophagy regulators and the use of reliable readouts to measure autophagic activities.

## 5. Modelling the role of autophagy in glioblastoma development

**Figure 2.**
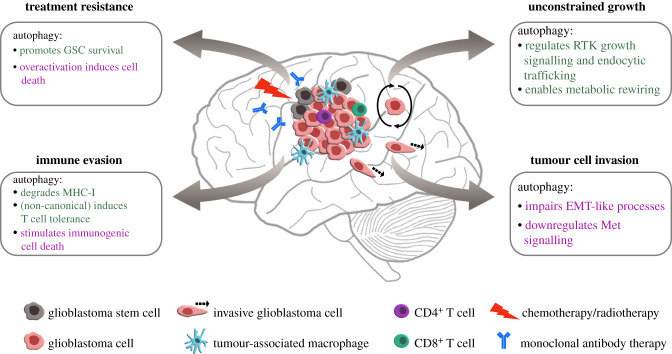
The potential relevance of autophagy in glioblastoma development and survival. Based on what is known from other solid tumour models and from findings in glioblastoma research, autophagy could be implicated in promoting (green text) or suppressing (purple text) various aspects of glioblastoma progression. Autophagy may support tumour growth by regulating receptor tyrosine kinase (RTK) signalling and trafficking, in addition to providing metabolites to fuel unconstrained proliferation. Autophagy may play a role in tumour cell invasion by regulating the epithelial-to-mesenchymal transition (EMT)-like process and limiting oncogenic Met signalling. Tumour cell immune evasion can be mediated by the degradation of major histocompatibility complex I (MHC-I) by autophagy, and immunosuppressive engulfment of dying tumour cells by non-canonical autophagy. By contrast, autophagy activation in dying tumour cells can stimulate immunity by releasing inflammatory signals. Upregulation of autophagy in response to treatment can support glioblastoma stem cell (GSC) survival while its overactivation potentially leads to tumour cell death.

The relevance of autophagy in glioblastoma is not fully uncovered. The properties of this invasive and resistant solid tumour could suggest a likely role for autophagy in promoting its growth (figure 2). Here, we will discuss published findings that are suggestive of the role of autophagy in glioblastoma survival.

### 5.1. Transcriptional evidence from glioblastoma patients

Assessing autophagy levels is one approach to examine its involvement in glioblastoma development. Enhanced autophagic activity evaluated by immunohistochemistry analyses of resected tumours has been shown to correlate with reduced glioblastoma patient survival [76,77]. Similarly, autophagy gene expression profiles in publicly available glioblastoma datasets show that the mesenchymal subclass exhibited increased *ATG* gene transcription, including *ATG7*, *LC3B, LC3C*, *ATG16L1* and *SQSTM1* (which encodes p62) [78]. In addition, genes that have been shown to induce autophagy, such as the p53-target, damage regulated autophagy modulator (*DRAM*), were also increased [78]. Interestingly, two recent studies have highlighted an association between high *ATG* gene expression signatures and reduced overall survival predominantly in patients with the mesenchymal subtype [79,80]. This is consistent with the mesenchymal subtype exhibiting worst patient prognosis [1]. Altogether, these findings suggest a correlation between enhanced *ATG* gene expression and a worse outcome for glioblastoma patients, thereby suggesting a potential role for autophagy in promoting glioblastoma growth.

### 5.2. Evidence from mouse models

As discussed previously, autophagy activation can impact tumour cells at various stages, including initiation, proliferation and response to treatment. A limited number of studies have examined how autophagy influences the formation of glioblastoma (table 1). An investigation modelling tumour initiation in mice demonstrated that, in the absence of autophagy, glioblastoma development is impeded [81]. This was achieved using the RCAS/TVA GEMM of glioblastoma induced by overexpressing *KRAS^G12D^* in a *Cdkn2a*^−/−^ background where autophagy was disrupted by genetic knockdown of *Atg7*, *Atg13* or *Ulk1* in tumour cells [81]. The molecular mechanisms underlying the requirement of autophagy in this GEMM remain unclear. Examination of *KRAS^G12D^*-expressing *Cdkn2a*^−/−^ glial cells in culture revealed that autophagy inhibition impaired anchorage-independent cell growth and induced senescence [81]. This model serves as a starting point to study autophagy in glioblastoma development as mutations in the *RAS* oncogene are only rarely detected in glioblastoma. Further GEMM studies using established glioblastoma oncogenic drivers are thus required to confirm the role of autophagy in initiating tumour development.

**Table 1.** Genetic inhibition of autophagy impairs glioblastoma development in mice (genetically engineered mouse model, GEMM; glioblastoma stem cells, GSCs).

model	autophagy inhibition	effects on tumour development	reference
RCAS/TVA GEMM (*KRAS^G12D^*, *Cdkn2a*^−/−)^	shRNA targeting: *Atg7, Atg13, Ulk1*	impeded tumour initiation	[81]
patient-derived GSCs xenograft	shRNA targeting: *ATG4B*	impaired tumour growth by GSCs expressing high MST4 levels	[82]

Autophagy can also implicate tumour cell proliferation. Orthotopic xenograft experiments of patient-derived GSCs in immunocompromised mice suggest a link between autophagy and levels of MST4 [82], a serine/threonine kinase that promotes cell growth and malignant transformation through activating ERK signalling [83]. ATG4B, one of four ATG4 isoforms required for the lipidation and delipidation of ATG8 proteins [34], is activated following its phosphorylation by MST4 [82]. Knocking down *ATG4B* impeded the growth of GSCs expressing high but not low MST4 levels [82]. It may be possible that GSCs with low MST4 expression are ATG4 independent due to potential compensation by other ATG4 isoforms supporting the processing of alternative ATG8 family members [84]. Interestingly, the high expression levels of *MST4* and *ATG4B* transcripts correlated with GSCs encompassing the mesenchymal-like molecular subtype and reduced patient survival [78,82], suggesting a potential molecular subtype-dependent role of autophagy in regulating glioblastoma cell growth.

Additionally, EGFRvIII-expressing glioblastoma cells, which are enriched in the classical subtype, have a greater dependency on autophagy for growth and survival during times of metabolic stress induced by nutrient-deprivation and hypoxia [85]. Non-specific autophagy inhibition by CQ treatment impaired EGFRvIII tumour growth in a heterotopic mouse model [85]. This was further translated to enhanced survival in CQ-treated patients with EGFRvIII-positive tumours compared to those expressing wild-type EGFR [85]. Altogether, these findings suggest a potential oncogene-dependent role of autophagy in promoting glioblastoma growth.

## 6. Autophagy in the tumour immune response

The interactions between glioblastoma and immune cells in the tumour microenvironment may have profound effects in determining treatment outcome. Manipulating autophagy has been described to be favourable in the context of immunotherapy, which includes dendritic cell (DC) vaccines, chimeric antigen receptor (CAR)-T cells and immune checkpoint inhibitors [86]. However, targeting autophagy during immunotherapy treatment in glioblastoma has not been studied. A pre-clinical glioblastoma study showed improved survival of mice following DC vaccination in combination with hypericin-based photodynamic therapy-induced immunogenic cell death [87]. Intriguingly, an autologous DC vaccination phase I clinical trial extended survival of glioblastoma patients by enhancing CD8^+^ T cell infiltration [88]. This improved survival was more prominent in patients predominantly harbouring the mesenchymal molecular subtype compared to those with the proneural subtype [88]. Since autophagy appears to be elevated in mesenchymal tumours (discussed above) and it can augment the immunogenicity of dying tumours by enhancing the release of the immunostimulatory signals (including calreticulin, HMGB1 and ATP) [89], it would be interesting to assess the contribution of autophagy activation in glioblastoma tumour cells in response to DC vaccination.

By contrast, autophagy may facilitate immune evasion, for example by degrading major histocompatibility complex (MHC)-I in tumour cells thereby reducing antigen presentation and CD8^+^ T cell recognition, as seen in PDAC [90]. Additionally, inhibiting autophagy by genetic or chemical inhibition of Vps34 in melanoma and colorectal cancer cells has been shown to promote their clearance in mouse models by triggering infiltration of inflammatory immune cells [91]. This enhanced the efficacy of immune checkpoint inhibitors targeting programmed death 1 (PD-1) and programmed death ligand 1 (PD-L1) [91]. It is therefore of interest to determine if autophagy inhibition can improve the outcome of recent PD-1/PD-L1 immunotherapy trials in glioblastoma patients [92–94].

Autophagy proteins not only play a role in the tumour cells themselves, but their function in immune cells can also contribute to the outcome of anti-tumour immune responses. The non-canonical lipidation of ATG8 proteins on single membranes is required in Lewis lung carcinoma myeloid cells to impair the local anti-tumour T cell activation in response to the engulfment of dying cells [95]. MHC-II antigen presentation by DC has also been shown to depend on non-canonical autophagy [49]. It remains to be investigated whether glioblastoma TAMs and DCs also use non-canonical ATG8 lipidation to deploy their inhibitory effects on anti-tumour T cells and promote tumour growth and invasion.

Further investigation and pre-clinical models are necessary to discover the role of autophagy in immune responses associated with glioblastoma and to distinguish the contribution of canonical versus non-canonical lipidation of ATG8 proteins. Particular care should be taken when considering if autophagy inhibition or activation will potentiate the immune response to target glioblastoma since the outcome may vary depending on if autophagy is manipulated in the tumour and/or immune cells.

## 7. Autophagy and glioblastoma invasion

The invasive nature of glioblastoma cells permits their infiltration to the surrounding normal brain tissue and hinders complete surgical removal. Tumour cells can acquire invasive properties by undergoing an epithelial-to-mesenchymal transition (EMT), a reversible process whereby cells lose epithelial cell properties and gain mesenchymal migratory characteristics. This process involves cytoskeletal remodelling, the loss of cell-cell adhesions and the degradation of the basement membrane and ECM [96]. EMT is transcriptionally regulated by the SNAIL family (SNAIL and SLUG), ZEB1 and ZEB2, and TWIST1. These transcription factors are activated by hypoxia as well as transforming growth factor-β (TGF-β) and RTK signalling pathways [96]. A characteristic of EMT is the downregulation of epithelial tight junction adhesion proteins, such as E-cadherin, followed by enhanced expression of mesenchymal markers including N-cadherin. This in turn stimulates Wnt signalling and the nuclear accumulation of β-catenin that activates the transcription of genes involved in proliferation and migration [97]. Intriguingly, glioblastoma cells have been found to activate an EMT-like programme [98]. In comparison to other malignant cells, the membrane localization of N-cadherin, rather than its expression, is reported to alter glioblastoma cell invasion [99]. Furthermore, reducing N-cadherin levels in glioblastoma cells has been shown to impair their focal adhesions and enhance their migratory capacity [100].

Autophagy has been shown to facilitate the degradation of SNAIL and SLUG in glioblastoma cells thereby consequently upregulating N-cadherin [101]. This suggests that autophagy may suppress glioblastoma invasive properties [100,102]. Furthermore, the Wnt pathway is particularly upregulated in a population of fast-growing, self-renewing proneural-like GSCs [103]. Autophagy has been previously shown to impede Wnt signalling by degrading a key component of the signalling cascade, Dishevelled [104]. Through this mechanism of Wnt signalling suppression, autophagy activation in glioblastoma cell lines can cause the relocalization of β-catenin to plasma membrane regions associated with N-cadherin [105]. These inhibitory functions of autophagy in glioblastoma invasion are further supported by the finding that autophagy stimulation by mTORC1 inactivation impaired the migration and invasion of patient-derived glioblastoma cells in culture [101].

Potential contradicting evidence exists that suggests the role of autophagy in supporting tumour cell motility and invasion [57]. Downregulating the autophagy players *DRAM1*, *SQSTM1* and *ATG7* impaired the capacity of patient-derived, mesenchymal-like GSCs to invade using a transwell cell culture system, supporting a role for autophagy in glioblastoma invasion [78]. In addition, autophagy has been documented to regulate oncogenic signalling of the RTK Met [106] which is amplified in approximately 4% of glioblastoma patients [107] and is associated with the invasive mesenchymal subtype [3]. In a xenograft mouse model of GSCs, Met overexpression was shown to support tumour invasion and resistance to monoclonal antibodies targeting the anti-vascular endothelial growth factor (VEGF) [108]. Activation of Met upon binding to its ligand, hepatocyte growth factor (HGF), can drive EMT and tumour metastasis [109]. Interestingly, optimal Met signalling requires non-canonical ATG8 lipidation on single membranes in various cancer lines [106]. On the other hand, autophagy may also suppress Met signalling by targeting its degradation through binding with LC3C in breast and cervical cancer cell lines [110]. HIF2*α* stabilization can reduce LC3C expression [111]; therefore, it could be hypothesized that enhanced expression of HIF in hypoxic tumours, such as glioblastoma, could release Met from LC3C-mediated downregulation [110]. Given that autophagy can both stimulate and inhibit Met signalling in various cancer cell lines, it would be interesting to determine how this regulatory network can implicate invasion of Met-expressing glioblastoma cells.

Overall, these studies suggest a role of autophagy in regulating glioblastoma invasion. However, it is important to take into account the effects of the tumour–stroma interactions and the tumour microenvironment (such as oxygen and nutrient availability) which can affect cell invasion [112]. More in-depth examination and the development of appropriate mouse models are therefore required in order to clarify the function of autophagy in the invasion of glioblastoma into the brain parenchyma.

## 8. Autophagy proteins facilitate receptor tyrosine kinase signalling

Inhibiting the RTK signal transduction pathways, which drive glioblastoma progression, is an attractive therapeutic strategy. Nevertheless, phase II clinical trials targeting RTKs, for example, tyrosine kinase inhibition of EGFR/EGFRvIII or PDGFR*α*/*β*, have failed to significantly improve patient survival compared to temozolomide or irradiation alone [113,114]. The ineffectiveness of small molecule inhibitors and antibodies targeting RTKs potentially occurs due to the development of various resistance mechanisms [115]. Therefore, finding alternative ways to clinically manipulate RTK signalling is required.

Accumulating evidence suggest an interaction between RTK signalling and autophagy [116]. Autophagy players can facilitate RTK signalling thereby potentially supporting the development of glioblastoma tumours that rely on RTK activation [106,117,118]. On the other hand, RTK signalling can suppress autophagy [66] and their chemical inhibition is widely known to activate autophagy in cells [116]. Given that RTK signalling is elevated in glioblastoma cells, it remains to be addressed how autophagy may still be active to support cell growth in the absence of RTK antagonists.

RTKs, such as EGFR, are activated upon binding to their ligands when located on the plasma membrane and continue to signal following endocytosis. Signalling from endocytosed RTKs can be either terminated upon targeting to lysosomes or maintained upon recycling back to the plasma membrane. Autophagy can regulate RTK signalling through various mechanisms. The loss of autophagy in immortalized glial cells has been shown to disrupt endosomal homeostasis, consequently perturbing EGFR trafficking and signalling in response to growth factor stimulation thereby reducing cell survival [117]. This was bypassed by EGFRvIII overexpression [117], with the difference in autophagy dependency likely to reflect its altered intracellular trafficking compared to wild-type EGFR [119]. Autophagic membranes can also be used as platforms to enhance MAPK signalling in response to EGFR stimulation [118] as has been shown with Met signalling [106]. In these settings, it would be interesting to test whether treatment with CQ/HCQ can affect EGF-induced signalling as inhibiting lysosomal activity does not disturb autophagic membrane formation and may stabilize EGFR.

While trafficking and signalling of EGFR is well studied thereby facilitating our understanding of its interplay with autophagy, PDGFR activation (associated with the proneural subtype) is less well understood. It remains unknown whether autophagy proteins play a regulatory role in PDGFR signalling and trafficking. Unlike autophagy inhibition by EGFR activation, autocrine PDGFR*β* signalling has been shown to enhance autophagy via stabilization of HIF1*α* during hypoxia [120], demonstrating a cross-talk between these pathways. Considering PDGFR*α* amplification is associated with 13.1% of glioblastomas [3], it would be beneficial to dissect how this receptor is regulated and whether inhibiting autophagy can suppress its activities.

## 9. Autophagy upregulation and resistance of glioblastoma cells to treatment

Glioblastoma tumours inevitably relapse and are resistant to temozolomide and radiotherapy treatment. To date, alternative strategies designed to suppress glioblastoma tumour cell growth have been unsuccessful. The reasons behind this include failure to target quiescent tumour-initiating GSCs, inherent plasticity of tumour cells, reduced drug delivery across the blood–brain barrier (BBB), and the lack of pre-clinical studies that model the complex interactions of glioblastoma tumour cells with the tumour microenvironment.

Quiescent stem cells are resistant to anti-cancer treatment that target actively proliferating cells by triggering cell death or inhibiting cell cycle progression. GSC heterogeneity and plasticity have contributed to the difficulty in translating drug responses observed in pre-clinical studies [2,8], which can potentially be resolved by the emerging new glioblastoma models [26]. These persistent GSCs can repopulate the tumour and result in relapse [7,121]. The recurring tumour exhibits reduced response to treatment potentially due to mechanisms like chemotherapy-induced hypermutation providing a selection pressure to favour the growth of resistant cells [121] or by upregulating survival mechanisms such as autophagy.

In addition to supporting the development of glioblastoma, it is recognized that autophagy is upregulated in response to the first-in-line treatment, temozolomide plus radiotherapy [69]. Autophagy can also be activated by RTK inhibition. For example, inhibiting EGFR (by erlotinib or ZD6474 treatment) or PDGFR*α*/*β* (by imatinib treatment) have been found to upregulate autophagy in glioblastoma cell lines [122–124]. The underlying mechanisms of how blocking various stages of autophagy can affect the survival of glioblastoma and other types of tumours remain to be further elucidated and have been reviewed elsewhere [70].

## 10. Manipulating autophagy to improve glioblastoma treatment

Accumulating pre-clinical studies suggest that autophagy inhibition enhances the sensitivity of glioblastoma cells to radiation [125] and chemotherapy including DNA damaging agents (such as temozolomide), histone deacetylase (HDAC) inhibitors and RTK targeting molecules [123,124,126–130] (table 2). Despite the lack of specific chemical regulators of autophagy, clinical trials targeting autophagy with lysosomal inhibitors (including CQ and HCQ) during combinational therapy are currently undergoing aiming to improve the outcome of currently used anti-cancer treatments [70].

**Table 2.** The effect of targeting autophagy on glioblastoma survival in response to treatment (3-methyladenine, 3-MA; bafilomycin A1, BafA1; Beclin 1, BECN1; chloroquine, CQ; hydroxychloroquine, HCQ; suberoylanilide hydroxamic acid, SAHA; temozolomide, TMZ; tyrosine kinase inhibitor, TKI).

treatment	autophagy modulator	treatment outcome	references
**clinical trials**			
surgery + chemotherapy + radiotherapy	CQ	prolonged patient survival	[131,132]
surgery + chemotherapy + radiotherapy	HCQ	no survival advantage	[133]
recurrent glioblastoma following surgery + chemotherapy + radiotherapy	rapamycin/sirolimus (mTOR inhibitor)	no survival advantage	[134]
recurrent glioblastoma following surgery + chemotherapy + radiotherapy	Rapamycin/sirolimus (mTOR inhibitor) + Erlotinib (EGFR TKI)	no survival advantage	[135]
recurrent glioblastoma following surgery + chemotherapy + radiotherapy	everolimus (sirolimus derivative) + Gefitinib (EGFR TKI)	no survival advantage	[136]
**mouse models**			
TMZ or SAHA	CQ	reduced tumour volume	[128]
ZD6474/vandetanib (multi TKI)	CQ	reduced subcutaneous tumour volume	[123]
**cell culture**			
TMZ	3-MA	promoted cell viability	[126]
	BafA1	enhanced cytotoxicity	
imatinib (PDGFR*α*/*β*, c-abl, and c-kit TKI)	si*ATG5* / 3-MA	promoted cell viability	[124]
	BafA1	enhanced cytotoxicity	
ZD6474/vandetanib (multi TKI)	sh*ATG7* / sh*BECN1* / CQ / 3-MA	enhanced cytotoxicity	[123]
SAHA	rapamycin	improved cell viability	[129]
	sh*LC3A* / sh*BECN1* / sh*ATG5* / BafA1 / CQ	enhanced cytotoxicity	
SAHA	sh*ATG7*	enhanced cytotoxicity	[130]

Although there is pre-clinical evidence that suppressing autophagy may be a promising mechanism to thwart resistance and enhance sensitivity to anti-cancer treatment, results from clinical trials have provided conflicting evidence [70]. Early trials reported enhanced survival following sustained CQ co-treatment with chemotherapy and radiotherapy in glioblastoma patients that had undergone tumour resection [131,132]. Conversely, in a more recent dose-escalation trial, administering daily doses of HCQ along with temozolomide failed to prolong glioblastoma patient survival [133]. The reason behind the discrepancy between these studies is not entirely clear but may be attributed to the high doses required for HCQ to inhibit autophagy following oral uptake, in comparison to CQ. This is a limiting factor, as demonstrated by reduced tolerance and increased toxicity [133]. Furthermore, the efficacy of CQ/HCQ in suppressing autophagy within the glioblastoma tumour mass remains questionable due to the unfeasibility to obtain multiple tumour biopsies from the brain. Researchers have therefore relied on testing autophagic activity in circulating peripheral blood mononuclear cells (PBMCs) from patients [133]. However, this may not reflect the status of autophagy in the glioblastoma tumour mass, which is likely to be exposed to lower doses of CQ/HCQ when compared to the circulating PBMCs (potentially due to the BBB or the acidic tumour environment) [137,138]. Developing alternative potent inhibitors that target autophagic degradation may be required to enhance anti-tumour efficacy.

Targeting HDAC activity is an emerging anti-cancer approach likely to exert its effects through the regulation of gene expression and chromatin remodelling [139]. Yet, a recent phase II clinical trial found no significant survival advantage for glioblastoma patients treated with the FDA-approved HDAC inhibitor suberoylanilide hydroxamic acid (SAHA) in conjunction with temozolomide and radiotherapy [140]. It is possible that autophagy activation in this setting contributed to the lack of enhanced survival advantage, which has previously been demonstrated in glioblastoma cell line cultures treated with SAHA alone [129] or in combination with temozolomide [128]. Pharmacological or genetic inhibition of autophagy in conjunction with SAHA treatment promoted cell death in cultured glioblastoma cells [129,130]. This has been promisingly replicated in a mouse model where combining SAHA or temozolomide with the non-specific autophagy inhibitor CQ significantly reduced tumour volume of glioblastoma cells orthotopically engrafted in C57BL/6 mice [128]. With this evidence, there is support for redesigning clinical trials with SAHA and CQ co-treatment to counteract autophagy-mediated tumour cell survival. Similarly, the clinical response to RTK inhibition could also potentially benefit from targeting autophagy. Using CQ to block autophagy induced by EGFR inhibitors can trigger cell death and impede tumour growth in mice [123]. In addition, impeding the last stage of autophagy by bafilomycin A1 treatment enhances anti-PDGFR*α*/*β* cytotoxicity in cell culture [124].

Inhibiting mTOR kinase activity has been used to treat various cancers. The inactivation of mTORC1 can directly induce autophagy by releasing the ULK1 complex from its inhibitory phosphorylation. In the case of glioblastoma, treatment with rapamycin, which inhibits mTORC1 by limiting its access to substrates, augments the cytotoxic response of irradiated primary glioblastoma neurospheres [141] and glioblastoma cells that were irradiated in combination with genetic silencing of EGFR [142]. These effects of inhibiting mTORC1 were shown to be mediated by autophagy activation, whereby blocking autophagosome formation chemically (by 3-MA treatment) bypassed the anti-tumour effects of mTORC1 inhibition [141]. Despite promising pre-clinical evidence for using rapamycin (sirolimus) or its derivative (everolimus) to treat glioblastoma patients, mTORC1 inhibition has failed to translate to effective clinical responses when used as a single agent [134] or in combination with EGFR tyrosine kinase inhibitors [135,136]. The lack of efficacy has been attributed to limited delivery to the tumour mass [134]. However, a more potent version of rapamycin coupled to an mTOR kinase inhibitor by a polyethylene glycol linker (RapaLink-1) has revealed glioblastoma tumour regrowth following preliminary regression in a mouse model, indicating that tumour cells eventually acquire treatment resistance to the cytotoxic effect of mTOR inhibition [143]. Whether activated autophagy contributes to the resistance phenotype needs to be investigated.

The disparity in the role of autophagy during glioblastoma response to therapy could indicate that the upregulation of autophagy beyond a specific threshold, for an example during rapamycin treatment, may cause intracellular stress that sensitizes cells to death-stimulating signals. Therefore, targeting autophagy as a means to treat glioblastoma requires careful investigation and modelling prior to clinical application.

## 11. Conclusion and future perspectives

Numerous findings have described the relevance of autophagy in tumourigenesis. Autophagy plays a role in maintaining cellular homeostasis and thereby prevents oncogenic transformation and tumour growth in certain tissues. On the contrary, it is also appreciated that cell stress-induced autophagy is used by many established tumours to enable their unconstrained growth in the restrictive tumour environment. Furthermore, autophagy upregulation in response to chemotherapy and radiotherapy can lead to treatment resistance by aiding tumour cell survival. To date, evidence from glioblastoma models indicates that autophagy is involved in tumour initiation, development and response to treatment. Yet manipulating autophagy in patients has had limited success in improving their survival indicating that understanding the relevance of autophagy in various aspect of glioblastoma requires in-depth investigation.

Currently, targeting autophagy in the clinical setting is limited to a few non-specific inducers and suppressors. For that reason, mechanistic understanding of autophagy requires additional research in order to develop novel and specific modulators. Additionally, it will be key to evaluate if autophagy dependency of glioblastoma tumour cells is influenced by molecular subtypes. Determining if a subset of patients is more likely to benefit from autophagy modulation could lead to personalized treatment and potentially better outcomes. Finally, given the impact of autophagy in both tumour and stromal cells, it would be important to employ whole-body genetic modulation of autophagy in pre-clinical studies in order to closely mimic its therapeutic targeting. It is possible that modulating autophagy in tumour and stromal cells may give opposing impact on improving survival.

## Data accessibility

This article does not contain any additional data.
